# Degenerative spinal stenosis may influence Normal Pressure Hydrocephalus (NPH)-related imaging characteristics

**DOI:** 10.1016/j.bas.2026.106279

**Published:** 2026-08-07

**Authors:** Hanna Böttner, Kerstin Jütten, Frederic DeBeukelaer, Klaus Radermacher, Anne Benninghaus, Christian Andreas Mueller, Hans Clusmann, Chuh-Hyoun Na

**Affiliations:** a Department of Neurosurgery, RWTH Aachen University, Pauwelsstraße 30, Aachen, 52074, Germany; b Department of Diagnostic and Interventional Neuroradiology, RWTH Aachen University, Pauwelsstraße 30, Aachen, 52074, Germany; c Chair of Medical Engineering, RWTH Aachen University, Pauwelsstraße 20, Aachen, 52074, Germany

**Keywords:** Craniospinal compliance, Normal pressure hydrocephalus, Spinal stenosis, CSF flow-dynamics, Callosal angle

## Abstract

**Background:**

The etiology of NPH is unknown, but might be related to altered craniospinal compliance caused by spinal canal narrowing, as has been suggested by in-vitro simulation studies. However, clinical evidence is lacking. We explored associations between cervical (CS) and lumbar (LS) spinal stenoses, and NPH-imaging characteristics.

**Methods:**

Spinal MRI of NPH-patients scheduled for shunt-surgery was retrospectively evaluated by measuring the minimal dural sac cross-sectional area (DSCSA), and anterior-posterior diameter (AP) in CS and LS. Cranial-CT was evaluated regarding Evans-Index, callosal angle (CA), and Radscale-Score. CS and LS were each sub-grouped by median split into small/large DSCSA and small/large AP, and CT-parameters compared using Mann-Whitney-U-Tests, tested one-sided (*p* < .05, Bonferroni-Holm-corrected for multiple comparisons *p*_Holm_). Partial correlations were explored between stenoses- and cranial-imaging parameters.

**Results:**

Out of 426 NPH-patients screened, 114 patients had spinal-MRI. Of those with cervical-MRI (n = 100), 90% had CS, of those with lumbar-MRI (n = 86), 71% had LS. Among these, 60 patients had cervical and lumbar MRI, of which 63% showed combined CS and LS. CA was larger in CS patients with small as compared to large AP (*p* = .045, *r* = .235, 95%CI [.002, .444]), and in LS patients with small as compared to large DSCSA (*p* = .022, *r* = .330, 95%CI [.060, .555]), which did however not remain significant after correction for multiple comparisons (*p*_Holm_> .05). Lumbar AP tended to be negatively correlated to the Radscale-Score (*p* = .041, *r* = −0.31, 95%CI [-.561,-.014], *p*_Holm_> .05).

**Conclusions:**

Spinal stenosis might be associated with NPH-related imaging characteristics. Prospective studies should investigate the potential pathophysiological impact of degenerative spine-disease on NPH.

## Highlights

• Simulation studies suggest spinal stenosis to impact on craniospinal compliance.
• We explored associations between spinal stenosis and NPH.
• Higher degree of spinal stenosis was associated with larger callosal angle.
• Degenerative spine disease may impact on CSF-hydrodynamics and NPH.

## 1 Introduction

Normal pressure hydrocephalus (NPH) is characterized by pathological intracranial accumulation of cerebrospinal fluid (CSF) in the brain, resulting in enlarged ventricles. It is a chronically progressive condition that primarily affects the elderly, leading to gait disturbance and ultimately immobility, dementia, and urinary incontinence (Adams et al., 1965; Vanneste, 2000). Population-based prevalence rates have been estimated to range from 0.2/100.000 in subjects aged 70-79 years, to 5.9/100.000 in those aged over 80 years (Jaraj et al., 2014). However, recent studies have reported significantly higher prevalence rates (Thavarajasingam et al., 2024), and a systematic survey suggests NPH prevalence to be even higher than generally assumed (Brean and Eide, 2008). This indicates that patients may often remain undiagnosed. Considering the demographic changes, with elderly subjects (>65 years) being the world wide fastest-growing age group expected to even outnumber people aged 15-24 years by 2050 (Chang et al., 2025), the prevalence of NPH is expected to increase dramatically in the coming decades, placing a greater burden on caregivers, and putting additional socioeconomic strain on health care systems. Therefore, improving the understanding of the pathophysiology of this condition and enhancing treatment outcomes for NPH patients is highly relevant.

Although the efficacy of CSF-diversion procedures has been demonstrated in numerous NPH patients (Pearce et al., 2024), shunt failure remains a prevalent occurrence (Williams et al., 1998), and disease progression continues unabated despite treatment. Unlike secondary NPH, which is caused by preceding or underlying pathologies such as hemorrhage or inflammatory conditions that alter CSF reabsorption and/or CSF flow-dynamics, the etiology of idiopathic NPH remains unclear. However, multiple factors have been suggested to alter CSF equilibrium (Das and Biagioni, 2025): These include an increase in CSF pulse pressure during blood pressure systole due to reduced CSF outflow and/or reabsorption, loss of the Windkessel effect in skull base arteries due to arterial hypertension, altered transependymal flow, and changes within the glymphatic system (Bubenikova et al., 2025). Decreases in CSF-efflux via the lymphatic system (Back et al., 2025) have also been reported as pathophysiological contributing factors. Furthermore, increasing evidence suggests, that the spinal compartment may be more relevant to NPH pathophysiology than is commonly acknowledged: Traditionally, it has been believed that CSF-reabsorption mainly occurs at the arachnoid villi as the interface between the subarachnoid and the venous space, mediating CSF-reabsorption along the pressure gradient between the two compartments (Bradley, 2015). However, it is also important to consider the role of CSF reabsorption and flow within the intraspinal compartment for maintaining intracranial CSF homeostasis (Maillot, 1991). In particular, the spinal compartment seems to be crucial for intracranial volume-pressure compensation, a process known as craniospinal compliance. Recent in vitro simulation studies provided evidence that intracranial CSF flow-dynamics and dynamic craniospinal compliance are altered by narrowing of the spinal canal (Benninghaus et al., 2026).

We therefore hypothesized that degenerative spine disease, which induces rigidity and narrowing of the spinal canal, may influence NPH by decreasing CSF reabsorption and altering CSF flow dynamics. However, there is a lack of patient data in support of this hypothesis. As a first exploratory step, we conducted a retrospective analysis of potential associations between cervical and lumbar spinal canal stenosis and NPH imaging characteristics in patients admitted for shunt surgery.

## 2 Materials and methods

### 2.1 Patient cohort

For this retrospective analysis, the database of a tertiary neurosurgical center was queried for patients with the ICD10 diagnosis G91.2 (idiopathic normal pressure hydrocephalus), planned for shunt surgery between 2013 and 2023. Patient data were found eligible for inclusion in the analysis if MRI of the cervical and/or lumbar spine prior to shunt surgery was available. Patients were clinically assessed using the NPH-grading scale score [Kubo et al., 2008], which scores cognitive, urinary and gait impairment, with higher scores indicating more severe clinical impairment and a maximum score of 12 points. The NPH-grading scale score was determined preoperatively and at follow-up (on average 976 days postoperatively).

### 2.2 Evaluation of spinal imaging data

**Fig. 1:**
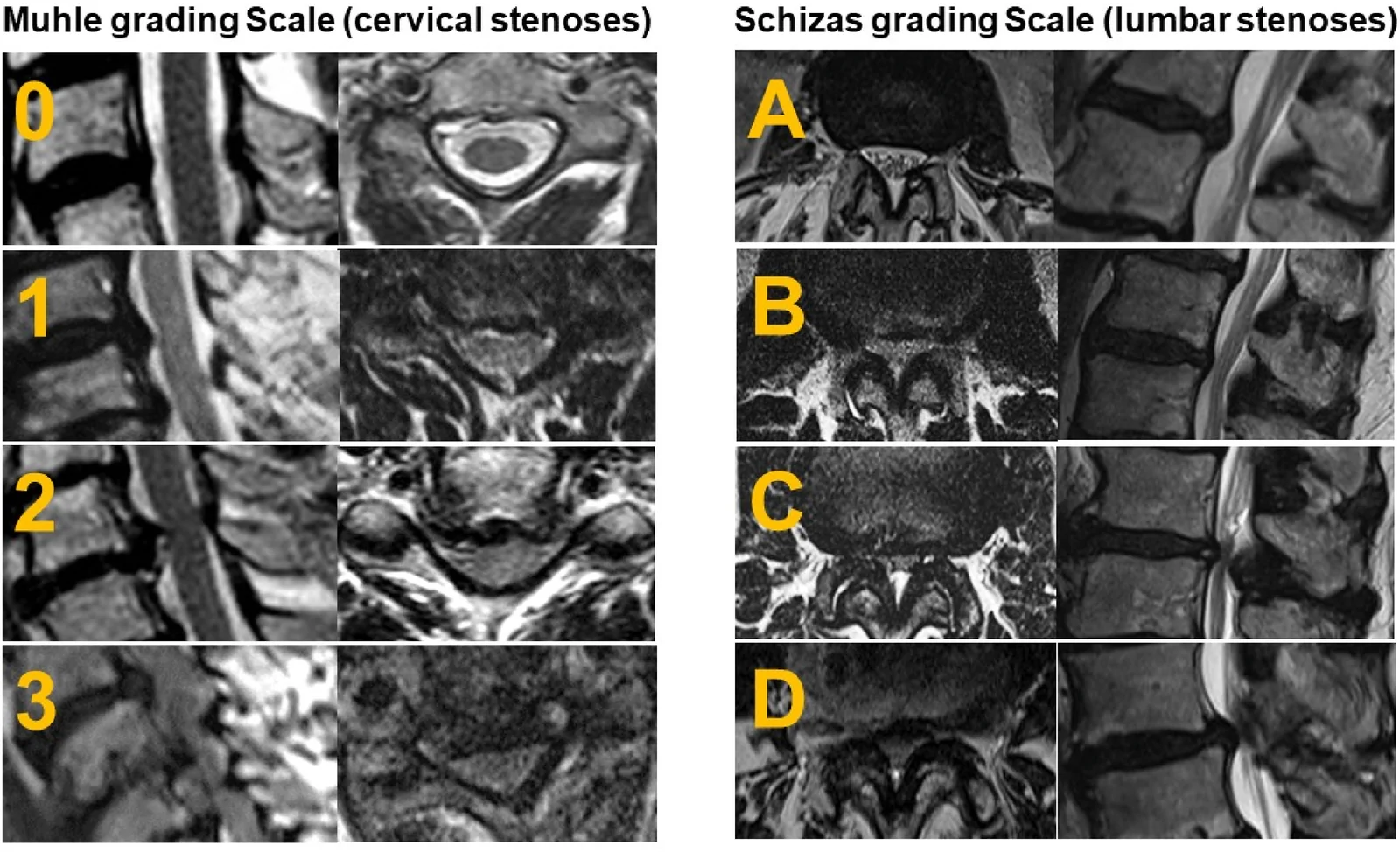
*Muhle grading scale* for cervical stenosis*:* Grade 0: no stenosis, 1: partial obstruction of the subarachnoid space, 2: complete obstruction of the subarachnoid space, 3: myelon impingement. *(Modified) Schizas grading Scale* for lumbar stenosis*:* Grade A: minor stenosis with CSF being clearly visible (AP diameter <13 mm), grade B: rootlets still discernible but CSF hardly visible, grade C: compressed rootlets without visible CSF, but epidural fat still visible, grade D: absolute stenosis with epidural fat being not/hardly visible.

Cervical MRI was evaluated by applying the Muhle Grading Scale (Muhle et al., 1998), which comprises grades 0 to 3. Grade 0 corresponds to a normal width of the spinal canal, grade 1 to partial obliteration of the subarachnoid space, grade 2 to complete obliteration of the subarachnoid space, and grade 3 to spinal cord impingement (Fig. 1).

Lumbar MRI was evaluated by using a modified version of the Schizas grading scale (Schizas et al., 2010), differentiating grades A to D, with grade A corresponding to minor stenosis (with AP diameter <13 mm according to Papanagiotou and Boutchakova, 2014]), but CSF being still visible within the dural sac. Grade B corresponds to moderate stenosis (with rootlets occupying the whole of the dural sac, but with single rootlets still being discernible), grade C to severe stenosis (with no singular rootlets or CSF within the dural sac being discernible, but with visible epidural fat posteriorly), and grade D corresponding to maximal stenosis (with no rootlets being discernible, as well as neither CSF nor epidural fat being visible) (Fig. 1).

The maximally stenotic segment in each patient was then quantitatively evaluated by determining the dural sac cross sectional area (DSCSA) in the axial plane (in mm^2^), and the anterior-posterior (AP) diameter in the midsagittal plane (in mm). In addition, the number of stenotic segments per patient, as well as frequencies per segment levels affected were analyzed.

### 2.3 Evaluation of cranial imaging data

Cranial computed tomography (CT) acquired prior to shunt-surgery was evaluated using the Radscale Score (Kockum et al., 2018), which is a composite score of 7 NPH-typical cranial imaging features comprising the Evans-Index, callosal angle, size of temporal horns, narrowness of high-convexity sulci, dilated Sylvian fissures, focally dilated sulci, and periventricular hypodensities. The Radscale-Score comprises maximally 12 points, with higher scores corresponding to more NPH-typical cranial imaging features and was previously found to correlate with NPH related symptom-burden (Kockum et al., 2018).

**Fig. 2:**
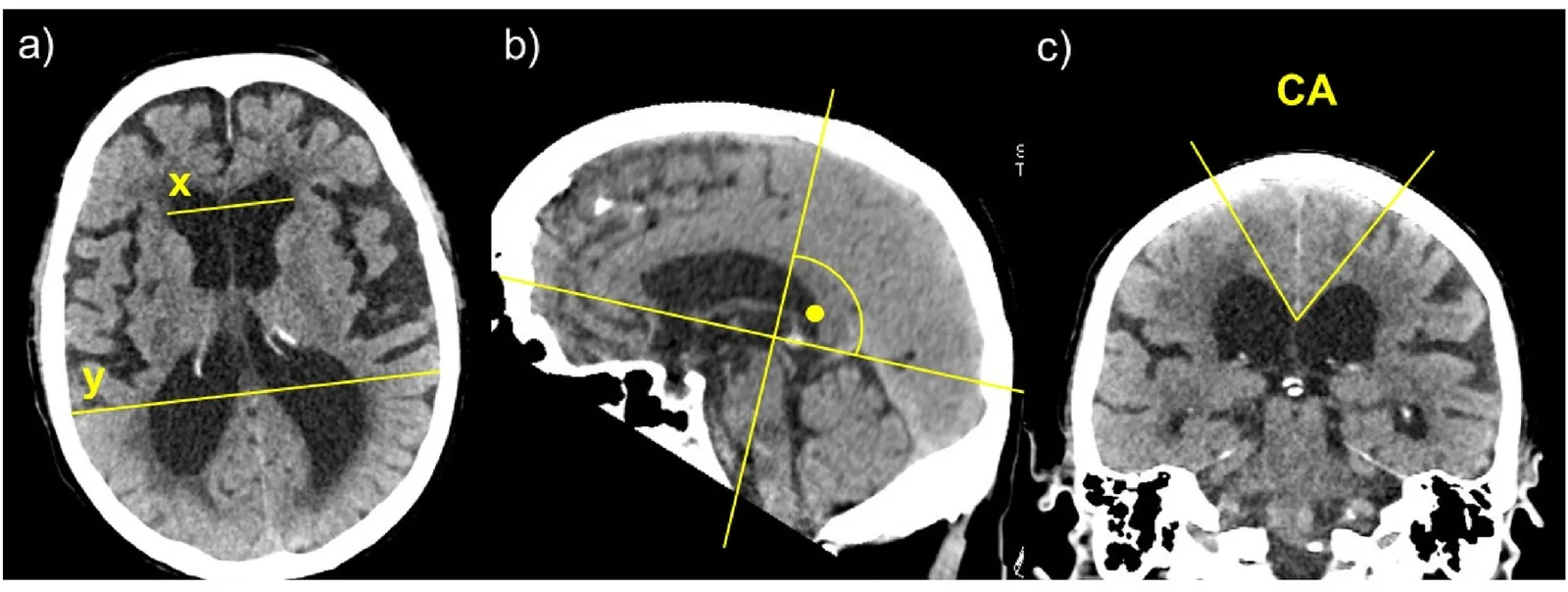
a) Evans-Index, defined as the maximal width of the anterior horns divided by the maximal intracranial biparietal width. Values > 0.30 indicate pathological enlargement of the ventricles. b) Callosal angle (CA) measured between the lateral ventricles in the coronal plane through the posterior commissure and orthogonal to the axial plane connecting the anterior to the posterior commissure (ACPC plane). CA is typically <90° in NPH patients.

In addition, absolute values of both, the callosal angle (measured in the coronal plane crossing the posterior commissure orthogonal to the axial plane connecting the anterior and posterior commissure), as well as of the Evans-Index (defined as the maximum width of the frontal horns divided by the maximum intracranial biparietal diameter, with values > 0.30 indicating pathological enlargement of the ventricles) were analyzed. CT data was only included, if preoperative imaging was digitally available and if multiplanar reformations were sufficient in image resolution (slice thickness ≤ 2 mm), see Fig. 2).

### 2.4 Statistical analysis

2.4.1

Descriptive statistics of patient demographics, number of stenoses, frequency of segments affected, and mean values of AP and DSCSA were determined.

All statistical analyses were performed with R [R Core Team (2022). R: A language and environment for statistical computing. R Foundation for Statistical Computing, Vienna, Austria. URL https://www.R-project.org/.], RStudio [Posit RStudio. Posit team (2024). RStudio: Integrated Development Environment for R. Posit Software, PBC, Boston, MA. URL http://www.posit.co/.], and Jamovi [The jamovi project (2022). jamovi. (Version 2.3) [Computer Software]. Retrieved from https://www.jamovi.org].

2.4.2

Hypothesizing, that patients would differ in hydrocephalus related cranial imaging parameters depending on the degree of spinal stenosis, patients with cervical stenosis (CS) and lumbar stenosis (LS) were each subgrouped based on their DSCSA by median split, resulting in subgroups with small/large DSCSA (CS: N_smallDSCSA_ = 45, N_largeDSCSA_ = 44, DSCSA cut-off = 85.7 mm^2^; LS: N_smallDSCSA_ = 30, N_largeDSCSA_ = 31, DSCSA cut-off = 75.0 mm^2^). For each subgroup analysis (small and large DSCSA subgroups, separately for LS and CS), the Evans Index, Radscale-Score and callosal angle were compared by applying Mann-Whitney U Tests, tested one-sided with a significance level of *p* = .05. Results were adjusted using the Bonferroni-Holm correction for multiple comparisons (k = 3 per family, *p*_Holm_).

2.4.3

In addition, group differences were analyzed accordingly for cervical (CS) and lumbar stenosis (LS) subgroups based on small/large AP diameters (CS: N_smallAP_ = 45, N_largeAP_ = 45, AP cut-off = 6.5 mm; LS: N_smallAP_ = 30, N_largeAP_ = 31, AP cut-off = 6.7 mm), comparing Evans Index, Radscale Score and callosal angle applying Mann-Whitney U Tests, tested one-sided with a significance level of *p* = .05. Results were adjusted using the Bonferroni-Holm correction for multiple comparisons (k = 3 per family, *p*_Holm_).

2.4.4

Partial correlations were computed in those patients in which both, cervical and lumbar MRI were available to assess associations between Evans Index, Radscale-Score, and Callosal Angle on the one hand, and cervical and lumbar AP-diameter as well as DSCSA on the other hand, while controlling for age. All resulting p-values were Bonferroni-Holm-corrected for multiple comparisons (*p*_Holm_).

## 3 Results

### 3.1 Patient demographics

A total of 426 NPH patients were screened, of which 114 had preoperative spinal MRI data available. 100 of these had cervical MRI, 86 lumbar MRI, and 60 of these patients had both, cervical and lumbar MRI. Mean age was 73 ± 10 years, and 53% were males (Table 1). In all patients planned for shunt surgery, previous spinal tap test or lumbar drainage had been proven to be therapeutically effective. Preoperatively, mean NPH-grading score was 6.38 ± 3.25, with significant improvement after shunt surgery (with a median follow-up of 926 days) to 5.75 ± 3.64 (*p* = .03).

**Table 1:** ) The first column on the left shows values across the whole cohort (n = number of patients). Columns 2 and 3 show values mean ± SD) of patients with CS and LS, respectively. *n = 33. Please note, that CS and LS patients do in part overlap, as some of the patients (n = 60) had both, cervical as well as lumbar imaging.

	Spinal MRI (n = 114)	CS (n = 90)			LS (n = 61)			
**Gender (m, f)**	60, 54	51, 39			34, 27			
**Age (years)**	73 ± 10	73 ± 10			74 ± 7			
**Evans index**	0.36 ± 0.07	0.36 ± 0.08			0.36 ± 0.07			
**Radscale**	8.04 ± 2.63	8.11 ± 2.55			8.40 ± 2.64			
**Callosal angle (°)**	67 ± 16	67 ± 17			67 ± 16			
NPH grading score (pre-OP)*	6.24 ± 3.44	6.24 ± 3.33			6.67 ± 3.26			
NPH grading score (post-OP)*	5.73 ± 3.58	5.40 ± 3.69			6.40 ± 3.2			
**Stenosis grade**	-	*Muhle 1:* 22	*Muhle 2:* 33	*Muhle 3:* 35	*Schizas A*: 18	*Schizas B*: 11	*Schizas C*: 22	*Schizas D*: 10

### 3.2 Descriptive statistics of spinal imaging data

#### 3.2.1 Cervical stenosis

**Fig. 3:**
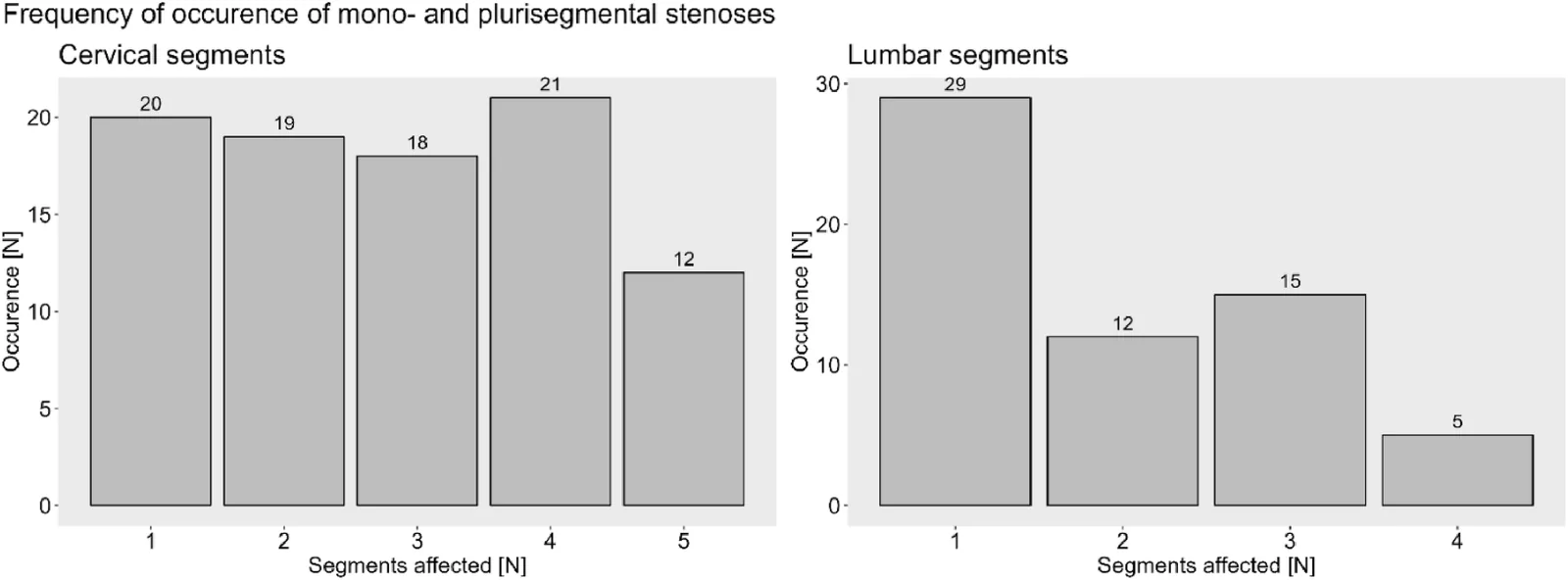
Frequencies of stenoses per segment level affected. On the left for cervical stenoses (CS), on the right for lumbar stenoses (LS).

Out of 100 patients with cervical MRI, 90 patients (90%) had cervical stenosis (CS), with the maximally stenotic segment corresponding in 22 patients to Muhle grade 1 (24%), in 33 patients to Muhle grade 2 (37%), and in 35 patients to Muhle grade 3 (39%). Mean AP of the maximally stenotic cervical segment was 6.4 ± 1.5 mm, and mean DSCSA was 89.1 ± 24.6 mm^2^. The cervical segments most often affected were C5/6 and C4/5 (for frequencies of segment levels affected see Fig. 3)

#### 3.2.2 Lumbar stenosis

Out of 86 patients with lumbar MRI, 61 patients (71%) had spinal lumbar stenoses (LS), with the maximally stenotic segment in 18 patients corresponding to Schizas grade A (29%), in 11 patients to Schizas grade B (18%), in 22 patients to Schizas grade C (36%), and in 10 patients to Schizas grade D (16%). Mean AP of maximal lumbar stenosis was 6.6 ± 2.6 mm, and mean DSCSA was 80.5 ± 38.2 mm^2^. Segment levels most often affected in the lumbar spine were L3/4 and L4/5 (see Fig. 3).

#### 3.2.3 Plurisegmental stenoses

**Fig. 4:**
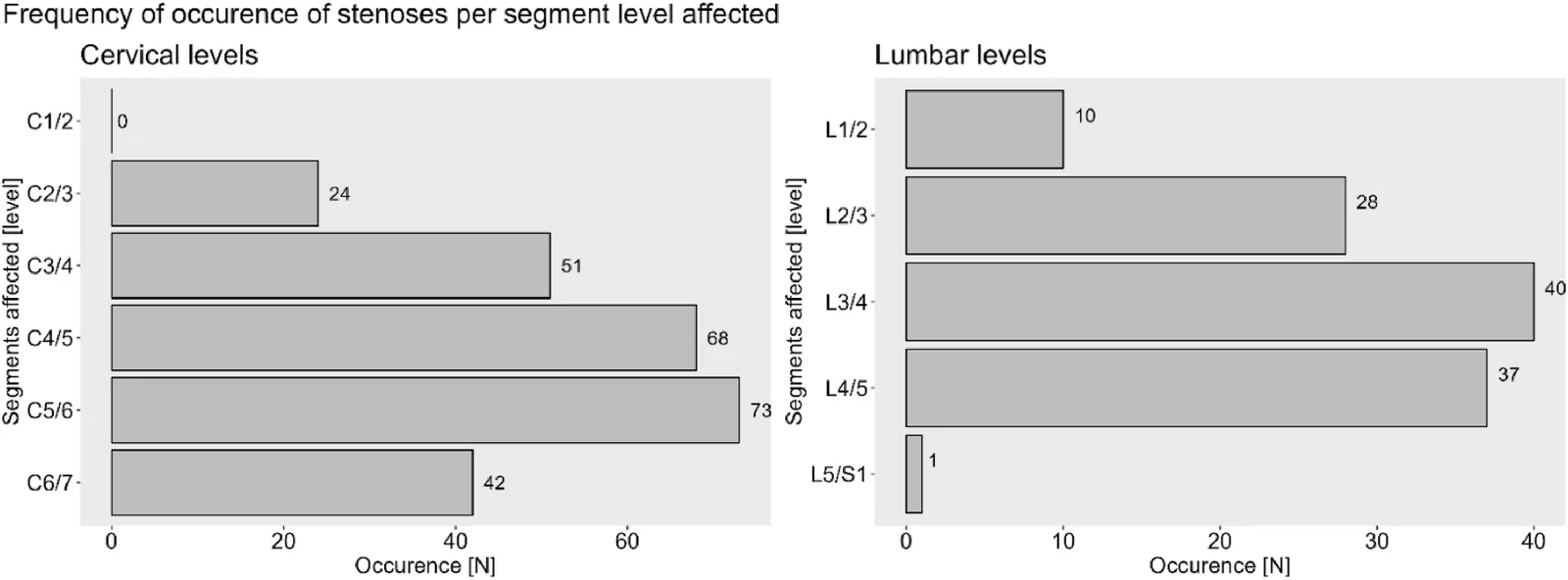
Number of patients with mono- or plurisegmental stenoses regarding the cervical spine (on the left) and the lumbar spine (on the right).

Plurisegmental stenoses, defined as the involvement of more than one spinal segment, appeared to be more frequent in the cervical spine as compared to the lumbar spine. 70 patients with CS (78%) had more than one cervical stenotic segment. 32 patients with LS (53%) had more than one lumbar stenotic segment. Of those patients with both, cervical and lumbar imaging (n = 60, 53% of the whole cohort), 3% had no stenosis, 25% had only CS, 8% had only LS, while the majority (63%) showed combined cervical and lumbar (dual-level) stenoses. For frequency of occurrence of mono- and plurisegmental CS and LS please see Fig. 4.

### 3.3 Descriptive statistics of cranial imaging data

For analysis of the cranial hydrocephalus-related imaging parameters, 85 preoperatively acquired cranial CTs were included in the analysis, showing pathological enlargement of the lateral ventricles with a mean Evans-Index of 0.36 ± 0.07, corresponding well to the diagnosis of NPH. The mean Radscale-Score was 8.04 ± 2.63, and the mean callosal angle was 67° ± 0.07, and thus well within the typically expected range (<90°) for NPH patients (For subgroup specifics please see Table 1.).

### 3.4 Subgroup comparisons

Group comparisons of imaging parameters between subgroups stratified by DSCSA and AP diameter, including significances for one- and two-sided testing, Bonferroni-Holm-corrected significances, effect sizes and 95%-confidence intervals (CI) can be found in Table 2.

**Table 2:** Group comparisons of imaging parameters between subgroups stratified by DSCSA and AP-Diameter.

Comparison	U	N (n^a^ vs n^b^)	*p*^1^ (one-sided)	*p*^2^ (two-sided)	*p* (Holm)^c^	*r*	95% CI	Effect size^d^
*Cervical*								
DSCSA small vs. large (Evans index)	636	39 vs 33	.468	.932	.854	.012	[−.221, .243]	very small
DSCSA small vs. large (Radscale)	573	39 vs 33	.210	.426	.630	.110	[−.125, .333]	small
DSCSA small vs. large (Callosal angle)	611	38 vs 33	.427	.854	.854	.026	[−.209, .257]	very small
AP small vs. large (Evans index)	644	38 vs 34	.511	.982	.511	.003	[−.229, .235]	very small
AP small vs. large (Radscale)	509	38 vs 34	.059	.122	.135	.212	[−.021, .423]	medium
AP small vs. large (Callosal angle)	481	37 vs 34	.045*	.088	.135	**.235**	**[.002, .444]**	**medium**
*Lumbar*								
DSCSA small vs. large (Evans index)	237	27 vs 24	.050	.101	.100	.269	[−.008, .507]	medium
DSCSA small vs. large (Radscale)	294	27 vs 24	.287	.571	.287	.093	[−.188, .359]	very small
DSCSA small vs. large (Callosal angle)	217	27 vs 24	.022*	.043	.066	**.330**	**[.060, .555]**	**large**
AP small vs. large (Evans index)	274	27 vs 24	.172	.345	.516	.154	[−.127, .412]	small
AP small vs. large (Radscale)	314	27 vs 24	.424	.850	.516	.031	[−.247, .304]	very small
AP small vs. large (Callosal angle)	287	27 vs 24	.242	.485	.516	.114	[−.167, .378]	small

Note.

U = Mann-Whitney U statistic; N = sample size per group (low vs. high), CT with insufficient resolution for multiplanar reformation were excluded; p = p-value; r = rank-biserial correlation, effect size for the Mann-Whitney *U* test; CI = confidence interval; DSCSA = dural sac cross-sectional area (mm^2^); AP = anteroposterior diameter (mm).

*p < .05, uncorrected.

a One-sided p-value as originally computed (intended, directional hypothesis).

b Two-sided p-value, shown for comparison.

c Bonferroni-Holm-adjusted p-value, based on the one-sided p-values; correction applied separately within each of the four families defined by region × split variable (cervical/lumbar × DSCSA/AP), each comprising k = 3 comparisons (Evans index, Radscale, Callosal angle).

d Effect size classification per Funder and Ozer (2019): effect size r = .05 = very small, .10 = small, .20 = medium, .30 = large, ≥ .40 = very large.

#### 3.4.1 Comparing CS patients with small and large DSCSA

In patients with cervical stenosis, DSCSA-subgroup comparisons did not reveal significant differences with regard to Evans-Index, Radscale Score, or callosal angle (all *p* > .05, *p*_Holm_ > .630) (please see Table 2).

#### 3.4.2 Comparing CS patients with high and low AP

Comparing CS subgroups based on AP-diameter did not show significant differences with regard to Evans-Index or Radscale Score (all *p* > .05, *p*_Holm_ > .135). However, subgroups differed regarding callosal angle, indicating higher mean callosal angle in patients with small as compared to large AP (callosal angle: medianAP_small_ = 70.7°, medianAP_large_ = 64.9°, *p* = .045), although this difference did not remain significant after correction for multiple comparisons (*p*_Holm_ = .135). Despite the lack of statistical significance after correction, the effect size was medium, r = .235, 95% CI [.002, .444] (please see Table 2).

#### 3.4.3 Comparing LS patients with small and large DSCSA

**Fig. 5:**
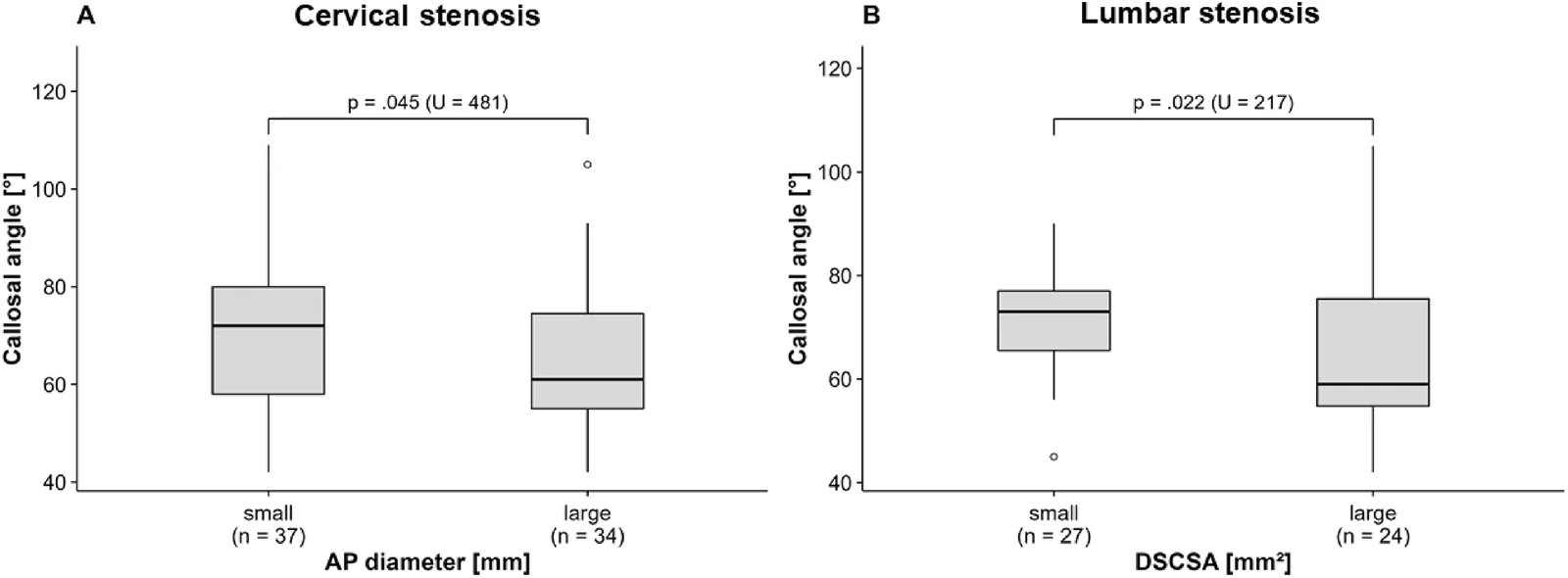
A) Comparison of the callosal angle between CS patients with small and large anterior-posterior (AP) diameter of the maximally stenotic cervical segment, and B) between LS with small and large dural sac cross sectional area (DSCSA) of the maximally stenotic lumbar segment (for further details please see Table 2).

In LS patients, small/large DSCSA subgroups differed neither in Evans-Index, nor Radscale-Score (all *p > .05*, *p*_Holm_ > .066). Results revealed however subgroup differences in callosal angle, indicating higher mean callosal angle in the small as compared to the large DSCSA subgroup (callosal angle: medianDSCSA_small_ = 71.0°, medianDSCSA_large_ = 64.3°, *p* = .022), although this difference did not remain significant after correction for multiple comparisons (*p*_Holm_ = .066, please see Fig. 5), effect size was large, r = .330, 95% CI [.060, .555] (please see Table 2).

#### 3.4.4 Comparing LS patients with small and large AP

Group analyses comparing LS subgroups with high/low AP revealed no significant differences in callosal angle, Evans-Index or Radscale Score (all *p* > .05, *p*_Holm_ = .516).

#### 3.4.5 Partial correlations

**Fig. 6:**
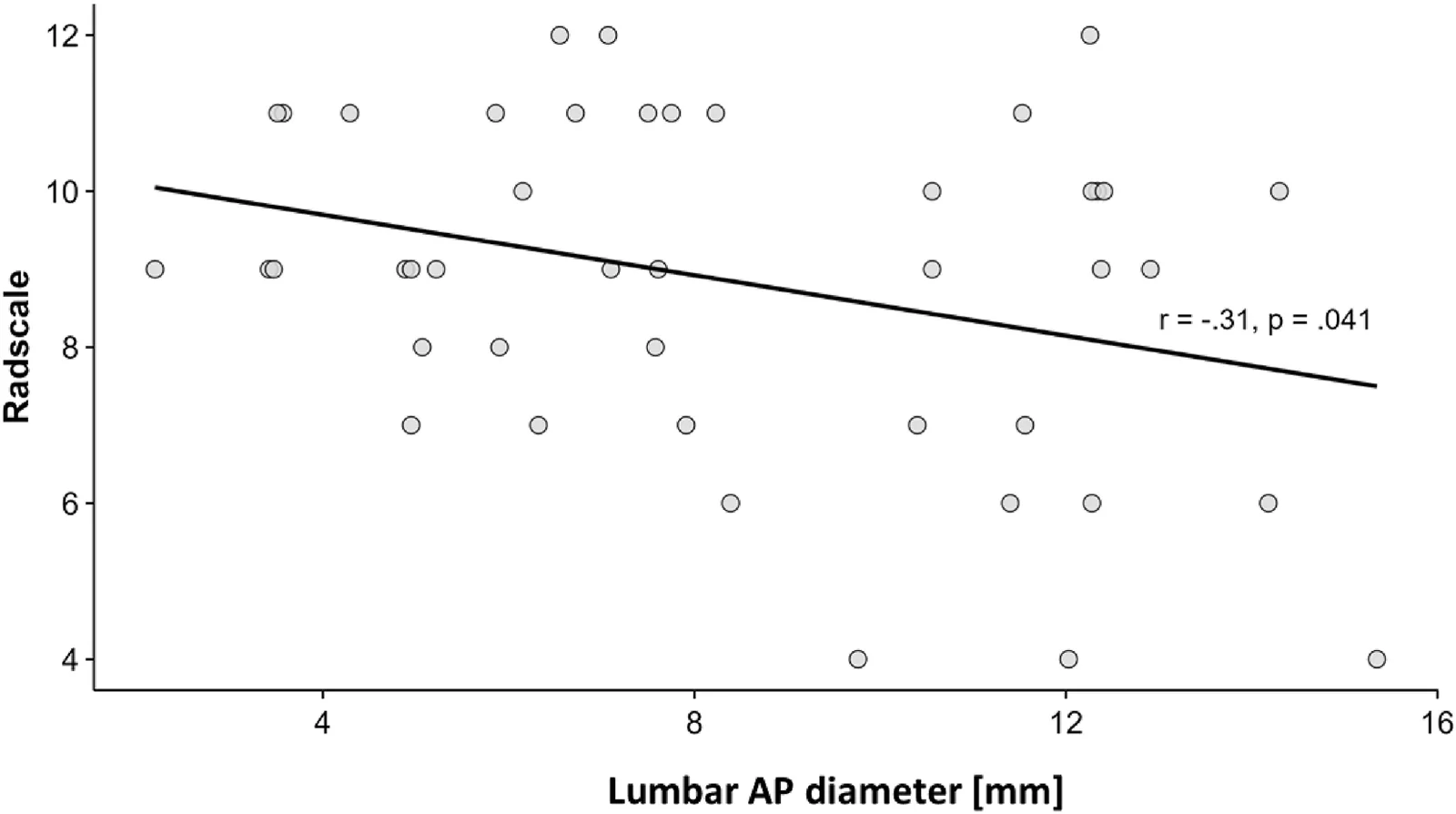
NPH-patients showed a trend for higher Radscale-Scores to be associated with smaller minimal lumbar AP diameter. The *p*-value is uncorrected, 95%CI [−.561, −.014]. Bonferroni-Holmes corrected *p*_Holm_ = .492 (for details please see Table 3).

Partial correlations, controlling for age, were computed between Evans Index, Radscale-Score, and Callosal Angle, and cervical and lumbar DSCSA as well as AP diameter. A significant negative correlation was observed between lumbar AP diameter and Radscale (*p* = .041, *r*_partial_ = −.31, 95%CI [−.561, −.014]) (please see Fig. 6). However, this association did not remain statistically significant after Holm correction (*p*_Holm_ = .492). All remaining partial correlations were non-significant both before and after correction (all *p* > .05, *p*_Holm_ = 1.000). An overview including partial correlations between spinal and imaging parameters, significances, effect sizes and 95% confidence intervals are given in Table 3.

**Table 3:** Partial correlations between spinal and imaging parameters.

Imaging parameter	*r*_partial_	95% CI	*p*	*p* (Holm)	Effect size^a^
*DSCSA, cervical*					
Evans Index	−.071	[−.364, .234]	.651	1.000	very small
Radscale	.135	[−.172, .418]	.387	1.000	small
Callosal angle [°]	−.091	[−.381, .215]	.561	1.000	very small
*AP, cervical*					
Evans Index	.024	[−.278, .322]	.879	.879	very small
Radscale	.204	[−.103, .475]	.189	.567	medium
Callosal angle [°]	−.130	[−.414, .177]	.407	.814	small
*DSCSA, lumbar*					
Evans Index	.073	[−.232, .365]	.642	1.000	very small
Radscale	−.139	[−.422, .168]	.373	1.000	small
Callosal angle [°]	.034	[−.269, .331]	.826	1.000	very small
*AP, lumbar*					
Evans Index	−.024	[−.322, .278]	.877	.877	very small
Radscale	**−.313**	**[−.561, −.014]**	.041*	.123	**large**
Callosal angle [°]	.142	[−.165, .424]	.362	.724	small

Note.

r_partial_ = Pearson's partial correlation coefficient, controlling for age (N = 44, df = 41 for all comparisons); CI = confidence interval; p = two-sided p-value; p (Holm) = Bonferroni-Holm-adjusted p-value, computed separately within each family (DSCSA/AP × cervical/lumbar; k = 3 comparisons per family: Evans Index, Radscale, Callosal angle); DSCSA = dural sac cross-sectional area (mm^2^); AP = anteroposterior diameter (mm).

*p < .05, uncorrected.

a Effect size classification per Funder and Ozer (2019): effect size r = .05 = very small, .10 = small, .20 = medium, .30 = large, ≥ .40 = very large.

## 4 Discussion

To the best of our knowledge, this is the first study to explore the relationship between degenerative spinal stenosis and hydrocephalus-related cranial imaging characteristics in NPH patients. Although our data cannot provide measures of craniospinal compliance or CSF flow dynamics, the associations found between spinal stenosis and NPH-related imaging features may support the hypothesis, that spinal canal narrowing promotes hydrocephalus formation through alterations of CSF hydrodynamics. Our data therefore seem consistent with findings of in vitro studies (Benninghaus et al., 2026), which recently demonstrated that cervical stenosis and lumbar stenosis increase CSF-flow resistance, resulting in a significant reduction in dynamic compliance and spinal CSF-flow. This led to an increase in intracranial pressure amplitudes (of up to 7.85 mmHg), which is believed to promote hydrocephalus formation (Eide, 2006).

In addition, a relationship between spinal canal measurements and ventricular size has recently been described even independent of NPH. Grosu et al. (2024) analyzed potential associations between the cervical spinal canal width and scoliosis, with white matter, grey matter and ventricular volumes. They found that a smaller width of the cervical spinal canal at the vertebrae level C2/3 was correlated with lower grey and white matter volumes, and a larger ventricular size. Based on these findings, authors suggested that spinal characteristics constitute independent risk factors for neurodegeneration, which is consistent with the results of our present study. Moreover, narrowing of the lumbosacral canal causing prestenotic slowing and reduced spinal CSF-flow (Kim et al., 2021) has previously been suggested as causative for an increase in CSF albumin quotient and protein concentration reducing CSF-reabsorption (Seyfert et al., 2002), which may also contribute to hydrocephalus formation.

In our cohort, the mean CA value (67° ± 16) was smaller than that reported for healthy subjects or patients with other neurodegenerative diseases in the literature. This value is therefore well within the expected range for NPH patients (Ishii et al., 2008). Such narrowing of the CA is considered a reliable biomarker for NPH, with a diagnostic accuracy of up to 93%, depending on the chosen threshold angle (Ishii et al., 2008; Baroncini et al., 2018; Miskin et al., 2017). Unlike healthy subjects, who typically exhibit larger callosal angles (mean 112° ± 11) than NPH patients, larger callosal angles observed in other neurodegenerative diseases (as e.g. in Alzheimer's disease: mean 104° ± 15) are indicative of e vacuo widening resulting from brain atrophy (Ishii et al., 2008). With CA having been described to be significantly smaller in NPH patients as compared to other neurodegenerative conditions (Ishii et al., 2008), those NPH-patients with CA in the expected range, but with relatively larger CA have previously been reported to be less responsive to CSF diversion treatment than those with relatively smaller CA. A cutoff value of 63° has been reported to have the best prognostic accuracy (Virhammar et al., 2014). This may suggests, that relatively larger callosal angles in NPH patients may be associated with secondary neurodegeneration and brain atrophy, and that symptoms are less likely to be reversible under CSF diversion. In our study, we found a trend towards larger CA in CS patients with smaller AP, as well as in LS patients with smaller DSCSA. As our analysis accounted for age, purely age-related effects were excluded. These findings therefore might support the hypothesis, that degenerative spinal stenoses chronically promote NPH by increasing CSF flow-resistance, reducing craniospinal compliance, and leading to higher intracranial CSF pressure amplitudes, which may promote secondary neurodegeneration in the long term.

However, some NPH patients may not exhibit an association between spinal canal narrowing and callosal angle, if neurodegeneration has not yet progressed, so that dynamic measures as such as CSF flow-velocity or CSF pulsatility would be required to actually capture the effects of spinal stenosis on intraventricular CSF hydrodynamics. It therefore might be, that NPH patients are more heterogenous in their associations between spinal pathology and static NPH imaging parameters, depending on the disease stage and the degree of secondary neurodegeneration, which could have contributed to the only borderline findings in our study. To validate this hypothesis, future studies are required that include larger patient samples and prospectively acquired imaging data such as dynamic CSF-flow measures (e.g. heavily T2-weighted constructive interference in steady state (CISS) sequences or cardiac-gated phase-contrast cinemode MRI of CSF), rather than only static imaging parameters.

NPH-grading scores significantly improved after shunt surgery, confirming a relevant degree of hydrocephalus-related symptoms responsive to CSF-diversion. However, it cannot be excluded, that spinal stenosis-related symptoms impacted the clinical presentation. A recent study by Tominaga et al. (2023) compared NPH patients with and without lumbar stenosis after shunt surgery and found, that those patients with coexisting LS experienced worse outcomes regarding gait functions. Therefore, coexisting spinal stenosis should be considered when assessing NPH patients and evaluating treatment outcomes. The prevalence of lumbar spinal stenosis in this cohort of 224 NPH patients, as previously reported by Tominaga et al. (2023), was 32.6%. However, based on the retrospective nature of our study and the incomplete spinal imaging data available, we were unable to determine the prevalence here. The prevalence of LS in the general population has been reported as ranging from 11 to 38% in the literature, but much higher prevalence have been found in older age groups (Jensen et al., 2020). It should also be noted, that the reported prevalence rate varies in the literature, as different studies have used different definitions for spinal stenosis. While some studies used only clinically defined criteria, others defined stenoses solely on the basis of radiological findings, whereas still others included only patients with symptomatic spinal canal stenoses (Kato et al., 2015; Jensen et al., 2020, Ishimoto et al., 2012). Future studies analyzing the prevalence of degenerative spine disease in NPH patients should use standardized spinal stenosis definitions based on radiological criteria. Determining the prevalence and characteristics of spinal stenosis in NPH patients could help to elucidate potential associations between degenerative spine disease and NPH. Therefore, larger epidemiological studies and the availability of nation-wide patient registries are needed. Taking into account concomittant degenerative spine disease in patients with NPH may also contribute to a better understanding of the substantial interindividual variability in responsiveness to the spinal tap test. Furthermore, those NPH patients undergoing surgical treatment for spinal stenosis should be longitudinally assessed to evaluate, whether spinal treatment might in the long term also positively impact on NPH disease dynamics.

### 4.1 Limitations

The major limitation of the present study is its retrospective nature, with spinal imaging data available for only a fraction of NPH patients screened. Therefore, selection bias must be taken into account, as those patients with spinal MRI scans may differ systematically from those without, which prevents generalizability of the present findings. Furthermore, statistical power was limited due to the heterogenous data structure with missing data, and only small sample sizes, which might have contributed to the only borderline findings of the present study. Although uncorrected analyses suggested significant effects, these were no longer significant after adjustment for multiple comparisons. Nevertheless, the corresponding effect sizes were consistently large, suggesting potentially robust underlying effects that warrant replication in larger samples. The limited sample size also prevented further analysis of the potential impact of specific spinal stenosis characteristics such as the level and number of segments affected or single versus multiregional involvement. A more detailed, systematic analysis of the potential impact of these characteristics on hydrocephalus, could therefore not be conducted.

Prospective acquisition of clinical and standardized total spine magnetic resonance imaging (including spinal canal volumetry with dynamic examinations using advanced MRI techniques to analyse cerebrospinal fluid pulsations and flow), as well as long-term follow-up with comparison to age-matched controls is therefore needed to investigate whether degenerative spine disease has an influence on NPH formation and disease dynamics.

While we recognise that the current study is only exploratory and rather hypothesis-generating than confirmative, we believe that focusing on the spinal compartment as a potentially relevant pathophysiological factor for NPH disease dynamics provides a novel and clinically highly relevant perspective that should be further pursued.

## 5 Conclusions

Spinal stenosis may be associated with NPH imaging characteristics, but these findings require validation in future studies with larger sample sizes. Prospective longitudinal studies should investigate, whether degenerative spine disease is pathophysiologically relevant to NPH.

## Ethics approval and consent to participate

The study was approved by the local ethics committee of the Medical Faculty of the University of the RWTH Aachen (CTC-A 22-089; EK106/22), and conducted in accordance with the standards of Good Clinical Practice and the Declaration of Helsinki. Patient consent was waived due to the retrospective nature of the study.

## Consent for publication

Not applicable.

## Availability of data and materials

The datasets generated and/or analyzed during the current study are not publicly available due to privacy protection obligations regarding patient data.

## Author contributions

Conception and design of the study: CN, KR, AB, HB.

Acquisition of data: HB.

Analysis and interpretation of data: HB, CN, KJ, FdB.

Drafting the article: HB.

Revising and editing the article: CN, KJ, KR, HC, AM, AB, FdB.

Final approval of the version to be submitted: HB, CN, KJ, KR, AB, FdB, HC, AM.

## Funding

The study was not funded.

## Declaration of competing interest

Authors hereby confirm that they have no competing interests in any way.
